# “It’s a Lot of Closets to Come Out of in This Life”: Experiences of Brazilian Gay Men Living with Human Immunodeficiency Virus at the Time of Diagnosis and Its Biopsychosocial Impacts

**DOI:** 10.3390/ejihpe14040070

**Published:** 2024-04-19

**Authors:** Felipe Alckmin-Carvalho, Henrique Pereira, Lucia Nichiata

**Affiliations:** 1 School of Nursing, Universidade de São Paulo, São Paulo 01239-020, Brazil; izumi@usp.br; 2 Department of Psychology and Education, Faculty of Social and Human Sciences, University of Beira Interior, Pólo IV, 6200-209 Covilhã, Portugal; hpereira@ubi.pt; 3 Research Centre in Sports Sciences, Health Sciences and Human Development (CIDESD), 5001-801 Vila Real, Portugal

**Keywords:** HIV/AIDS, sexual and gender minorities, HIV-related stigma, homosexuality, qualitative study

## Abstract

We investigated the experiences of Brazilian gay men with HIV, focusing on the moment of diagnosis and its potential biopsychosocial impacts. This clinical–qualitative study involved 15 participants interviewed online and synchronously by a clinical psychologist in 2021. Thematic analysis was employed to analyze the data. Interpretations were grounded in Minority Stress Theory. Four thematic axes emerged, including “Diagnostic Revelation”, “Social and Internalized Stigma”, “Biopsychosocial Effects of Living with HIV”, and “Gratitude for Treatment Advances and the Brazilian Health System”. The diagnosis was often experienced as traumatic, exacerbated by the absence of empathy and emotional support from healthcare providers. Participants commonly reported guilt, fear upon learning of their HIV status, social isolation, loneliness, lack of social support, and damage to affective-sexual relationships. Many also noted a decline in mental health, even those without HIV-related medical complications. Despite over 40 years since the HIV epidemic began, the prevalence of homophobia and serophobia among gay men remains widespread, including within the multidisciplinary teams of specialized services. This indicates that the stigma associated with homosexuality and HIV persists, despite significant biomedical progress in the diagnosis and treatment of the infection, particularly in Brazil.

## 1. Introduction

Infection with human immunodeficiency virus (HIV) is a complex condition that progresses slowly and, if left untreated, leads to the development of acquired immunodeficiency syndrome (AIDS) [1]. The disease was first recorded in mid-1977 in the United States, Haiti, and South Africa. However, it was not classified as a new disease until 1982 in San Francisco, in the western United States [1]. The first case in Brazil was diagnosed in 1982 in São Paulo [2].

In Brazil, approximately 1.2 million people live with HIV, with many others infected but unaware of their status [3]. The gay male population is disproportionately affected by HIV [3,4]. An epidemiological study conducted in 10 Brazilian capitals evaluated the serology of 3384 MSM [5]. The data collected in 2009 revealed an HIV infection rate of 14.2% (95%CI: 12.1–16.6%) [5]. A subsequent study by the same team, using identical methods, was conducted in 12 Brazilian cities [6]. In 2016, the serology of 3958 MSM was assessed, showing a national average HIV prevalence of 18.4% (95%CI: 15.4–21.7%). São Paulo, Brazil’s largest urban area, had a prevalence of 24.8% (95%CI: 18.5–32.4%), indicating that about one in four men in the city live with HIV [6]. These findings demonstrate significantly higher HIV rates among MSM compared to the general population, underscoring the need for targeted research.

HIV infection is now a chronic condition [7]. Biomedical advances, such as self-testing, enable early diagnosis, while the development of highly effective and low-toxicity antiretrovirals allows most individuals diagnosed with HIV to maintain good physical health and enjoy a high life expectancy, as identified in various countries [8,9,10,11]. In Brazil, since 1996, Law No. 9313/96 has ensured the free distribution of antiretroviral drugs through the Unified Health System (SUS), making it the first developing country to adopt a public policy for access to antiretroviral treatment, a move that has been globally recognized [2].

Recent studies conducted in developed countries indicate that men living with HIV have life expectations comparable to their non-HIV-infected peers, particularly those diagnosed early and consistently adhering to antiretroviral therapy [7,11,12]. It appears that the primary physical health challenges for Brazilian gay men with HIV who have access to treatment have been surmounted due to advancements in care for individuals with this condition. Nonetheless, the stigma surrounding homosexuality (homophobia) and HIV (serophobia) remains a significant issue in Brazil [13,14,15].

Even though the Brazilian Federal Supreme Court criminalized homophobia in 2019, the phenomenon remains widespread throughout the country [16]. For instance, a recent study found a high level of homophobia perceived within the Brazilian community and internalized by participants [17]. Over 93% believed that Brazilian society punishes homosexual individuals, and 98.55% acknowledged that discrimination against homosexual people is still prevalent in the country. Additionally, more than 22% are uncomfortable contemplating their sexuality, and 22% prefer to engage in anonymous sexual partnerships. These alarming rates of internalized and perceived homophobia underscore the urgent need for intersectoral actions in Brazil to reduce stigma.

Alckmin-Carvalho et al. [13] investigated the perception of internalized and community serophobia among gay men living with HIV. The study found that participants commonly perceived serophobia, both within the community and internally. The majority reported concealing their seropositivity due to the associated risks and ostracization by the Brazilian community. Additionally, societal prejudice persists, with the general population often viewing HIV as an infection exclusive to gay, bisexual, transsexual, and transvestite men, particularly those with “promiscuous” sexual habits [18]. A recent Brazilian study highlighted the surprise and perceived immunity to HIV infection among heterosexual men upon diagnosis [19].

In a Brazilian study, Costa et al. [20] assessed the perception of stigma associated with HIV among 1784 diagnosed individuals. The sample, which was diverse in gender and sexual orientation, reported experiences of discrimination by health unit employees based on their serological status while seeking HIV care in the past 12 months. The study found that 15.21% had encountered discrimination, with a higher likelihood among gay, bisexual, transgender, and nonbinary individuals, those deprived of liberty, sex workers, and drug users, compared to the general population (OR = 1.77; 95% CI [1.30–2.42]). These results underscore the amplification of HIV-related stigma in the presence of other marginalized identities.

Although some national quantitative studies have investigated the implications of an HIV diagnosis on the quality of life, self-esteem, and psychopathologies of gay men, this study is among the first qualitative inquiries into the biopsychosocial impacts associated with communicating the diagnosis to Brazilian gay men living with HIV in the era of “Highly Effective Antiretroviral Therapies” and “Undetectable Viral Loads”, as it has been conducted in other more developed [21]. We contend that qualitative studies are instrumental in thoroughly understanding the dynamics of the HIV epidemic in Brazil and its challenges within specific populations, such as gay men.

Understanding the modes of suffering and the nature of the physical and mental burden experienced by users of health services specializing in HIV/AIDS can enhance the empathy of professionals in the interdisciplinary team when providing care to this population. A more welcoming and well-informed attitude and reduced stigma promote better relationships and communication between patients and health professionals. This, in turn, contributes to treatment adherence and improved prognoses. Therefore, we believe this study can be beneficial for psychologists, psychiatrists, nurses, infectious disease specialists, social workers, pharmacists, and other health professionals who care for gay men living with HIV. Our objective was to investigate the experiences of Brazilian gay men living with HIV, focusing on the moment of diagnosis and its potential biopsychosocial impacts.

## 2. Materials and Methods

This clinical–qualitative study is particularly valuable in health contexts, as it facilitates a deep understanding of individuals’ meanings and narratives associated with the experience of illness [22,23]. It encompasses a range of clinically relevant topics, including the individual’s understanding of the disease and its causes, the biopsychosocial impacts currently experienced and anticipated in the future, and perceptions of the treatment and healthcare team.

### 2.1. Selection of Participants

This article focuses on the qualitative aspect of the project titled “Adherence to Antiretroviral Treatment, Psychopathologies, and Quality of Life of Men Who Have Sex with Men and Living with HIV”, with a particular emphasis on the mental health of key populations affected by HIV. The project employed a mixed-methods design. During the initial quantitative phase, researchers explored the impact of homophobia and serophobia on quality of life and mental health indicators among gay men with HIV [9,13,17]. The psychological assessment instruments were completed online and anonymously by 138 Brazilian gay men diagnosed with HIV. Data collection for this quantitative study was initially conducted in person at an institution specializing in HIV/AIDS diagnosis and treatment within the Brazilian public health service. However, due to the COVID-19 pandemic, which saw high rates of community transmission and deaths in Brazil from April 2020 to January 2022, the format was shifted online.

In the quantitative phase, participants were recruited through snowball sampling [24]. This method was chosen because, even 40 years after the onset of the HIV/AIDS epidemic, finding participants in Brazil to discuss topics such as HIV, homophobia, serophobia, and mental health is still difficult. Five researchers affiliated with the postgraduate Nursing and Public Health program at the master’s and doctoral levels and working in public institutions dedicated to the care of individuals with HIV/AIDS were selected as initial seeds. Sociodemographic and clinical data of the 138 participants in the quantitative study can be found in Alckmin-Carvalho et al. [13].

After the quantitative study’s online protocol, participants were asked the following question: “Would you be willing to be interviewed by a clinical psychologist via videoconference to discuss your experiences related to HIV?” Those willing to participate were instructed to provide their email addresses.

The inclusion criteria included being Brazilian, having lived in Brazil for most of their lives, self-identifying as gay, living with HIV/AIDS, being diagnosed with HIV/AIDS, and being over 18 years of age. The exclusion criteria were self-identifying as bisexual (this inclusion criterion was used because we consider that there are many differences in social dynamics related to stigma regarding bisexual men, which would justify a new study to seek in-depth understanding of the particularities of the impact of the diagnosis exclusively in this population) or as any sexual orientation other than gay, lacking a private place to discuss sensitive topics, and not having a stable internet connection.

Of the 138 participants who responded to the quantitative form, 36 agreed to participate in the clinical interview phase and provided their email addresses. The researcher conducted the interviews individually to assess the theoretical saturation criterion [25,26], which was used to determine the sample size. It was concluded that after 15 interviews, additional discussions on the experiences of gay men with HIV would yield minimal new data. Only one participant declined to have the interview recorded and was consequently excluded from the study.

### 2.2. Clinical Interview

The interview was conducted using a semi-structured script designed for this study. The first author, a 36-year-old cisgender, gay, white Brazilian man of medium-high socioeconomic status, led the process. The interviewer, a clinical psychologist with 13 years of experience, holds a Ph.D. in Clinical Psychology from a Brazilian public university.

The research question that guided this study was “In what ways has the HIV diagnosis impacted the lives of Brazilian gay men and how does it influence life in the present?”. The clinical–qualitative interviews were conducted in the latter half of 2021 using a semi-structured script specifically developed for this study. Participants were asked the following questions: (1) The diagnosis of HIV is often a sensitive moment for many individuals. Could you please describe your diagnostic experience in as much detail as possible? (2) In what ways has your HIV diagnosis affected your life? (3) How do you manage your HIV diagnosis at present?

### 2.3. Procedures

The interview was scheduled for a day and time convenient for the patient and was conducted synchronously using Google Meet. The interviewer introduced himself as a Brazilian gay man deeply interested in the dynamics of LGBTQIA+ rights. He then established a rapport by engaging in a few minutes of pleasant conversation about the city the interviewee mentioned and other everyday topics, aiming to create a neutral or even pleasant atmosphere. 

Subsequently, participants provided sociodemographic information, including age, skin color, education, occupation, income, and marital status, as well as clinical information, such as the date of HIV diagnosis, presence of an AIDS diagnosis, and current health status. The assessment of current health status was categorized into two possibilities, as follows: (a) symptomatic, when the patient reported acute or chronic diseases, whether associated with HIV/AIDS at the time of the interview or not and (b) asymptomatic, when the patient did not report any acute or chronic diseases at present.

The rapport and participant identification phases were intentionally not recorded to minimize individual exposure and enhance the likelihood of discussing sensitive topics. Participants were informed that recording would commence following the completion of the sociodemographic questionnaire and at the start of the clinical interview questions. The confidentiality of the research and the methodological choice to record were emphasized. Participants could select between a video call with audio and visual or an audio-only recording. The length of the interviews was not preestablished. Participants did not receive financial incentive for participating in the study.

### 2.4. Data Analysis

The interviews were transcribed, and participants’ names were coded to prevent recognition. Following transcription, the recordings were deleted. Both the first and second authors independently transcribed and analyzed the interviews. They conducted the qualitative analysis using the thematic analysis technique [27]. The authors collaboratively developed analysis categories and subcategories, which had not been predetermined. They worked together to resolve any disagreements about emerging themes. No software was utilized to assess the content.

To analyze the emerging themes, we anchored the study in the Minority Stress Theory [28], which provides a comprehensive understanding of the social determinants associated with the high prevalence of mental health issues in socially discriminated and marginalized populations, such as gay men living with HIV. Empirical studies have shown that these populations experience disproportionate stress from early development. Specific social stressors, including serophobia and homophobia, compound the everyday stressors affecting the general population, heightening individuals’ vulnerability to the development and exacerbation of mental health problems.

### 2.5. Ethical Considerations

The research project received approval from the Human Research Ethics Committee of the University of São Paulo School of Nursing in accordance with Brazilian legislation. The process number is 4,601,952, CAAE: 31527820.7.0000.5392, dated April 2021. All participants provided their signatures on the free and informed consent form.

Following the interview, the researcher proposed additional meetings within a psychotherapeutic framework for the subsequent weeks should the participant feel weakened, distressed, or anxious from recalling delicate or potentially traumatic topics associated with their HIV diagnosis and life. Only one participant (P1) sought psychotherapeutic support. Four sessions were conducted, after which the participant was formally referred for ongoing psychotherapeutic monitoring and psychiatric evaluation at a public mental health unit in their city.

## 3. Results

Among the fifteen participants interviewed, two (P2 and P7) opted for audio-only video calls; P2 cited an unstable internet connection, while P7 preferred not to have their image recorded. The interviews, lacking a predetermined average duration, ranged from 30 to 90 min.

The sociodemographic and clinical profiles of the 15 selected participants were notably diverse. For instance, P4 possessed a high level of education and income, was diagnosed early during the acute phase of HIV infection, and experienced no health complications related to AIDS. Conversely, P1 was a low-income individual with limited education, was diagnosed in the advanced stage of AIDS, and was suffering from life-threatening opportunistic diseases. Despite these disparities, most participants were aged between 30 and 40 years, identified as white, belonged to low or medium socioeconomic brackets, held undergraduate and/or postgraduate qualifications, were single, asymptomatic, and had been diagnosed with HIV less than 10 years prior. Seven of the fifteen participants received less than R$2900 per month, which classified them as low class (D and E) according to the criteria of the Center for Social Policies of Fundação Getúlio Vargas (CPS/FGV). Another seven participants had a monthly income greater than 2.9 salaries and less than R$22,000, being classified as middle class (B and C). Only one participant had an income greater than R$22,000 per month, being classified as high class (A).

All participants reported an undetectable viral load in their most recent blood test and consistently adhered to antiretroviral therapy. Table 1 outlines the sociodemographic and clinical characteristics of the participants.

Four categories emerged from the analysis of the interview corpus, which are listed and discussed below, including (1) diagnostic revelation and its effects; (2) social and internalized stigma; (3) biopsychosocial effects of living with HIV; and (4) gratitude for advances in HIV treatment by the Brazilian health system.

### 3.1. Diagnostic Disclosure

Generally, interviewees described the moment of receiving their diagnosis as distressing, frightening, and traumatic. Even those armed with current information about HIV and its treatment and who knew others living with the virus felt a sense of initial alienation upon diagnosis, which then gave way to worry, fear, anxiety, and guilt. Psychological support, acceptance, and a compassionate approach from the healthcare team delivering the diagnosis appeared to alleviate the negative effects of this revelation.

I knew the information, but at the time of diagnosis, we forget everything and think we are going to die, both our body from the disease and our soul from so much loneliness.

*I’m lucky that I had an enlightened nurse who told me about the diagnosis. She was very affectionate and had a calm voice. I remember her face to this day. It was an angel that God placed in my life. She works there [in the health service] to this day. She made a lot of difference in my life. About three months after [the diagnostic revelation], I took her a little gift to thank her for the way she treated me*.

(P5)

*The infectious disease doctor gave me the diagnosis. I found it very firm, but a good firm [way]. He calmed me down and said everything correctly, and I felt confident in his style. He was a very intelligent doctor. I felt like I was in good hands and that he wasn’t judging me. He had a welcoming manner*.

(P11)

*I discovered HIV in 1999, more than 20 years ago, during a blood donation campaign. The social worker who came to talk to me about the diagnosis was very empathetic, very calm, and conveyed confidence. It was an angel. It sounded like a mother talking to her son*.

(P3)

Conversely, some participants recounted negative experiences with health professionals’ attitudes and conduct upon revealing their HIV diagnosis, potentially exacerbating the trauma of the event. They criticized the insufficient privacy, the hurried nature of the consultation, the precipitous commencement of bureaucratic processes for treatment program enrollment, and the disregard for their emotional suffering and need for support.

*The day of diagnosis was terrible. The girl [referring to the health professional] spoke to me with the door open, she never asked if I was ok. It looked like a robot carrying out a task. She was on autopilot. I thought she had no humanity. “Humaniza SUS” is beautiful in theory, but in practice, it doesn’t work*.

(P7)

Humaniza SUS is a Brazilian federal government program that has existed since 2003 to implement the principles of the SUS in daily care and management practices, qualifying public health and encouraging solidarity exchanges between managers, workers and users. The program prioritizes valuing the subjective and collective dimension in all care and management practices in the SUS, strengthening the commitment to citizenship rights, highlighting the specific needs of gender, ethnic-racial, sexual orientation/expression and specific segments (black, rural, indigenous people, quilombolas, homeless population, among others)” [29].

*I did not have any psychological support at the time of diagnosis. The nurse called me, saying that I had to go there [to the HIV diagnosis and treatment unit of the Brazilian public health system] to retake the exam. I did it again, and the next day, the doctor called me. I went into his room, he handed me the test and said: you have HIV, and he started talking a lot about the treatment, about what I should do, and paperwork to fill out. There was a lack of welcome, it seems. I had no emotional support. After he talked about the diagnosis, I didn’t hear anything else. The appointment didn’t last 15 min, but it felt like an eternity. There was a lack of humanity. I know that HIV nowadays isn’t the same thing it used to be if you take the medicine properly, but it’s not the flu*.

(P2)

### 3.2. Social and Internalized Stigma

Nearly all participants described encountering homophobia and serophobia, whether overt or insidious, perpetrated by healthcare professionals, particularly doctors. The interviewees said these professionals declined to administer essential treatments or make pertinent medication adjustments.

*I had an episode of prejudice once with a doctor who was going to operate on my nose because I have an adenoid. When he found out I had HIV, he didn’t want to operate on me anymore. He canceled the surgery without giving any reason. Of course, the secretary didn’t tell me that, but I’m not stupid. I noticed the prejudice. That’s why I don’t talk to anyone. Nowadays, I don’t even talk to doctors. If a doctor is already terrified, imagine normal people*.

(P14)

Another participant attributed the attitude and clinical management of a medical professional from a service specializing in HIV diagnosis and treatment to homophobia. This was based on the professional ’s rude manner during care and his refusal to change a medication that caused the interviewee to experience a highly uncomfortable adverse event.

*I remember that a while ago, I took that medicine [for HIV] that made my eyes yellow. And a lot of people know what that meant. And at the time I was a waiter. I was very embarrassed. I asked the doctor a lot to change my medicine, but he did not want to change it. The nurse said there was a way to change it, but he said there was no way. He was older, probably around 60 years old. I think he didn’t like gay people, he was a bit rough. I was terrified of suffering prejudice at work, of people being disgusted with me, so I stopped taking the medicine for a month. Nothing happened, but I thought it would be better to go back*.

(P15)

Other participants reported the negative impact of disclosing their diagnosis on various aspects of their lives, including romantic relationships and work. These impacts encompassed not only serophobia but also violations of their right to confidentiality concerning their HIV serological status.

*After the diagnosis, I told my husband. He did not accept. We ended a 10-year relationship. I was in the process of being fired from the company I worked for. I asked HR [the company’s human resources department] to keep the health plan for a while longer. I thought I might need it because of HIV. I told them my diagnosis. They accelerated my dismissal, and my HIV diagnosis was leaked to a company of over 4000 employees. I was a small fish, a nobody. I was isolated, ashamed, and alone. But I sued them, and after two years, I managed to be reinserted into this company and received a high amount, as if I had worked all the time I was away. God was dozing but woke up at the end*.

(P5)

Reports indicating the prevalence of both internalized homophobia and internalized serophobia among participants were also common. For instance, P7 disclosed that, before his HIV diagnosis, he harbored negative perceptions of individuals living with the virus.

*I thought it was dirty to have HIV. I already had contact with a heterosexual couple who had HIV from church, and everyone knew. I remember going to their house, even, but I was afraid of drinking water there, of getting too close. And look what life is like. Now I have HIV, and I think people will feel that way with me too—this fear, this repulsion*.

(P7)

For this participant, his pre-diagnosis internalized prejudice about people living with HIV influenced his perception of the community’s stigma concerning his condition. Patients frequently described HIV as an emotional burden, which, compounded by homophobia, complicated daily life and the formation and maintenance of relationships. Additionally, some participants experienced social and internalized stigma, demonstrating the bidirectional relationship between these phenomena.

*I’m already gay, and I still have HIV. Is very bad. It’s too much. People cannot know one thing or another. Otherwise, I’m lost (P7). I am part of a very important voluntary service within the Catholic Church. There have already been several accusations inside that I am gay. They didn’t expel me—I think they turn a blind eye. Now, if they know that I have HIV, then there’s no way. I will definitely be expelled and isolated. Very few people know about my serology, and I want it to stay that way*.

(P7)

*I thought HIV was very far from my reality. I had the impression that those with HIV were promiscuous people who had many partners. For me, the diagnosis was very impactful*.

(P2)

*After knowing that my health was not at risk, that my lymphocyte count was good, and that the viral load was undetectable, I started thinking about social issues, you know? Wow, sometimes being gay and having HIV is tiring. It’s a lot of closet for us to come out of in this life. First, homosexuality, which is a blow from childhood, then you come out of the closet and deal with fear, rejection, and low self-esteem. When you think you are more stable in adult life, HIV comes along. Another closet for us to go out. Tired. But I move on*.

(P15)

Among the reported behaviors and feelings characteristic of both perceived community serophobia and internalized serophobia, the most frequent included fear of discovering an HIV diagnosis due to potential social ostracism, shame regarding the HIV diagnosis, and the compulsion to conceal antiretroviral medication.

*I’m very afraid that people will find out that I have HIV. The medicine is a burden for me. It’s ugly, something I have to hide. It’s like an ugly relative that we don’t want to show to anyone. I hide my medicines in the car. In fact, I’m ashamed that people find out that I take this medicine for HIV*.

(P11)

In participant P2′s narrative, the link between homophobia and serophobia within the community becomes clear. P2 perceives HIV as an additional socially stigmatized trait, compounding his identity as a gay and feminine man. He believes this combination exacerbates the challenges he faces in forming affective-sexual relationships.

*HIV was really bad for me. It would be better to live without this virus. I’m already gay, effeminate, and I still have HIV. Very difficult to find someone [referring to the emotional-sexual partnership]*.

(P2)

### 3.3. Biopsychosocial Effects of Living with HIV

We observed that the biopsychosocial effects of living with HIV varied significantly based on several factors: the life stage at which the diagnosis occurred, the stage of HIV infection (initial/intermediate vs. AIDS phase), the socioemotional skill set present at the time of diagnosis, the duration since the HIV diagnosis, preexisting knowledge about HIV, the degree of serophobia (both internalized and socially perceived), and the extent to which the social support network was established.

Overall, mental health deteriorated, with increased isolation and the emergence or exacerbation of psychopathologies. Participants also experienced feelings of helplessness, loneliness, isolation, fear, hopelessness, and a sense of loss, even when the HIV infection was recent and asymptomatic, lacking significant clinical complications. Additionally, many participants found it increasingly difficult to resume daily activities after diagnosis.

*I had a lot of depression. I lost a lot of weight. I didn’t recognize myself anymore. The diagnosis [of HIV] took me off track. I had many plans, many dreams. The diagnosis of AIDS and everything that came after [referring to four hospitalizations for treatment of opportunistic diseases] took me off track. It was a lot of suffering. I didn’t have a relationship with anyone for a year. You take the HIV medicine, but the medicine doesn’t treat your head.*

(P8)

Thirteen of the fifteen participants did not reveal their HIV diagnosis to their family of origin, nor did they consider that the members of the nuclear family would be capable or emotionally available to offer support and affection. Three participants did not mention the nuclear family. This hesitation in revealing the diagnosis in the family can be seen in P7′s speech:

*I was raised in an Evangelical context; everything was a sin. My family is very prejudiced. I never told them anything about my homosexuality. I’m not going to tell them about HIV either. After HIV I felt even more alone. I don’t have many friends. I’m a more closed person. Now more closed for fear of prejudice. There was a time when I was so alone that I even thought about killing myself*.

(P7)

Among the most prevalent psychosocial effects was the negative impact on participants’ affective-sexual lives, with many beginning to avoid opportunities for emotional bonding and sexual encounters due to fear of disclosing their diagnosis and the risk of HIV transmission.

*After HIV I felt a lot of shame and fear of passing it on to someone [referring to HIV transmission]. I didn’t even like kissing on the mouth. I was alone for a long time. I couldn’t relate. One of the few times I tried, I broke down out of fear. Then it got even worse. Then I started taking everything for friendship, never for flirting.*

(P5)

*I will always have this issue of telling the diagnosis. My sex life is very inactive. It was already difficult before [the HIV diagnosis]. Then it got worse. I stay alone to protect myself*.

(P8)

Most interviewees were diagnosed with HIV during the initial or intermediate stages, reporting little to no negative repercussions on their physical health. However, seven participants received their diagnoses late, presenting with immune system impairment consistent with AIDS. Of these, only two were symptomatic at the time of the interview, including P1, due to complications still associated with AIDS (owing to the inability to restore the immune system despite an undetectable viral load), and P14, who experienced conditions typical of premature aging in HIV patients, such as hypertension and diabetes. These participants endured significant suffering related to life-threatening opportunistic diseases, extended hospital stays, social isolation, and financial losses. For patients diagnosed with AIDS from 2020 onward, like P1, the aforementioned challenges were compounded by the fear of COVID-19 infection, which poses a greater risk to immunocompromised individuals.

*I discovered very advanced HIV. I had already destroyed my defenses [referring to irreversible changes in the immune system]. Even taking the medicine correctly, my defense cells did not return to normal. I get very sick. My health got much worse. Before I was normal. After HIV, I’ve had Kaposi’s Sarcoma, neurosyphilis, cytomegalovirus and many other opportunistic infections. In 2021 I spent more than three months in the hospital. Totally isolated because of COVID-19. I thought about killing myself many times. Every month I have a health problem—tooth infection, lung problem, heart problem. I still have AIDS, because my defenses have not recovered. I no longer make plans for the future, even in the short term. Now I only have the present. I’m not sure of anything. It’s like living on an eternal tightrope. I see myself as a time bomb. I don’t know when I’m going to explode*.

(P1)

Over half of the participants indicated that their HIV diagnosis catalyzed enhancing their self-care practices. Reported improvements included adopting healthier lifestyle habits, such as ensuring proper nutrition, establishing a regular physical exercise routine, managing sleep patterns, and moderating the use of alcohol and other substances.

*After HIV, I started to look more closely at my health. I like to live. I know the medicine saves me. I stopped drinking a lot, I’m exercising more, eating better. I now need to improve my sleep, but my health is better*.

(P6)

*My physical health has changed for the better. Completely changed. Sleep, cholesterol, food. Everything got better. I continue smoking and drinking my beer, but physical exercise has improved a lot. I cycle and swim. Before, I didn’t do anything. I also never took a blood test to check my health. Now I do it twice a year*.

(P5)

*My health has improved a lot after HIV because I take better care of myself. I replaced vitamin D and B12. I stopped smoking and started drinking much less. I started walking more hours a week and taking more care of my diet. I started eating more Omega-3. I was already careful, but after HIV I became even more careful. And the good part was that I already knew, being in the health field, what an acute HIV infection was like. So, I made an early diagnosis and started treatment very quickly. There was no time for the virus to change my immune function. Before HIV I had a throat infection every year, I took antibiotics and everything. After I started exercising, sleeping better, and eating better, I never had a bacterial infection again*.

(P4)

*I take care of myself more now. We mature. This diagnosis comes and we are afraid of dying, of having serious problems. I stopped using marijuana and “padê” [referring to cocaine]. I still drink a lot, but only beer. I stopped using distillate drinks*.

(P11)

Thus, it has been confirmed in the preceding sections that enhancing self-care skills resulted in improved physical health following an HIV diagnosis, except in cases identified during the AIDS phase.

### 3.4. Gratitude for Advances in Treatment by the Brazilian Health System

Speeches expressing gratitude were confirmed regarding biomedical advancements in HIV treatment, particularly concerning the reduced number of pills, the increased effectiveness of medication in controlling the virus, and the diminished adverse effects. This sentiment was more pronounced among individuals diagnosed and treated over extended periods, exemplified by P3 and P14, who have managed the virus and its treatment for 23 and 26 years, respectively.

*Felipe [referring to the interviewer], I used to take more than 20 pills. Do you know what this is? To have pills for lunch? It was an endless sadness. I took it and cried. Today I only take two. It’s a wonderful thing*.

(P14)

Feelings of gratitude were also linked to the awareness that maintaining an undetectable viral load over time and consistently adhering to medication prevents HIV transmission, even during unprotected sex. The concept “undetectable = untransmissible” was spontaneously mentioned by eight of the fifteen participants interviewed. This knowledge was associated with a marked decrease in the fear of transmitting the virus during sexual encounters. It lessened the perceived need to disclose an HIV diagnosis to casual sexual partners. Consequently, this had a positive effect on the sexual well-being of most interviewees.

*This undetectable thing, which has happened in recent years, has helped me a lot because now I can have sex without fear with whoever I want, and as I’m not putting the person’s life at risk, I don’t think I have to tell them. I know it because my doctor said so. This undetectable thing is a serious thing, research that has already been done all over the world. It doesn’t really happen. It was my salvation. I was terrified of passing it on to someone, so I wouldn’t have sex, and people would lose patience and leave. They didn’t understand why I didn’t want to have sex. After the undetectability, I started having sex again. It was too good*.

(P12)

*I have always trusted science. So much so that I went on to do a PhD in my field. Now, in this COVID-19 health crisis, I was trying to convince my family, who are all Bolsonarists [referring to Brazilian President Jair Messias Bolsonaro, a Brazilian far-right politician who discouraged Brazilians from getting vaccinated during the COVID-19 pandemic]. When I heard about the Partner studies, which concluded that undetectable equals untransmittable, I was very happy. I deeply thank the researchers who helped us with this. It’s a way to stop the AIDS epidemic, of course, but in personal terms, for me, it meant reclaiming my sexuality. I just don’t like that expression: [interviewee’s name] is undetectable. I am not undetectable. I’m still here. It is the virus that is undetectable*.

(P15)

Participants also expressed contentment with the Unified Health System (SUS), which adheres to principles of comprehensive health care and universality in serving Brazilians. Most participants particularly valued the free distribution of medication. Those who had observed the progression of treatment methods since the onset of the AIDS epidemic, like P14, and participants with higher educational levels, such as P4–a professional in biomedicine familiar with advancements in the field–acknowledged the significance of these developments.

*The SUS is very good. It provides my medicines for free. And they are very good medicines, the same ones used in Europe. I know because I’m a biomedical professional, and I did some research. Dolutegravir, which is one of the ones I take, is excellent. I didn’t have any side effects from the medicine. I only took Tenofovir off because it caused kidney problems. I was left with just Dolutgegravir and Lamivudine on a simplified combination. I feel a lot of gratitude for the SUS*.

(P4)

## 4. Discussion

Our study aimed to thoroughly comprehend the experiences of Brazilian gay men living with HIV, focusing on the moment of diagnosis and its potential subsequent biopsychosocial impacts. We found that the diagnosis was often perceived as a potentially traumatic event, particularly when coupled with a lack of empathy and emotional support from healthcare providers. Common experiences among participants included guilt, fear upon learning of their HIV status, social isolation, loneliness, and detrimental effects on affective-sexual relationships related to HIV.

Participants generally reported a high perception of serophobia and homophobia within the general population and among health professionals, including those in Brazilian health services specializing in the care of people living with HIV. This observation aligns with the findings of a recent Brazilian study [20], which indicated that marginalized minorities, such as gay men with HIV, were about twice as likely to experience serophobia in specialized Brazilian health services for people with HIV compared to non-marginalized groups.

Conversely, these findings contrast with those from a Portuguese qualitative study [21], in which none of the 31 interviewed gay men living with HIV reported discrimination in HIV treatment units. We posit that episodes of serophobia from healthcare team members have a particularly detrimental impact on individuals with HIV. One consequence of such behavior is clear, which is a diminished trust in disclosing their diagnosis to other health professionals and an escalated fear of sharing their HIV status with their social connections. This results in losing opportunities to obtain emotional support from positive disclosure experiences.

Studies suggest that the potential negative mental health impacts associated with receiving a positive HIV diagnosis and managing the virus’s psychosocial consequences can be mitigated by a robust social support network [29,30,31]. Researchers have also noted the beneficial effect of such support networks on adherence to antiretroviral treatment [32] and the consistent reduction of viral load in gay men living with HIV [33]. However, the research found that nearly all interviewees shared a desire to conceal their serological status to avoid moral judgments related to sexual practices or social orientation, as well as individual blame for HIV infection. This lack of social support was evident given that none of the 13 participants, including the youngest, felt comfortable disclosing their diagnosis to nuclear family members or even close friends. The hesitation to reveal an HIV diagnosis and the absence of social support, which increases an individual’s vulnerability and exposure to stigma-related stress, was also observed in the study by Pereira, Caldeira and Monteiro (2018) [21]. The authors identified ambivalent feelings like those in the present study; on the one hand, there is the desire to disclose the HIV diagnosis to potential romantic partners, friends, and family members, for social and emotional support, and on the other hand, there is the fear of rejection, the risks of disclosure, and the potential for moral judgment and blame. 

In our study, most interviewees deemed it crucial to maintain the confidentiality of their HIV status. Consequently, many withdrew from romantic relationships to prevent disclosure or to minimize the effort required to conceal their condition. This behavior aligns with findings from other national [13] and international studies [34,35]. For instance, a Brazilian cross-sectional study assessing internalized serophobia among 138 gay men living with HIV found that a significant majority (72–95%) endeavored to keep their HIV diagnosis secret. They expressed regret over any previous disclosures, perceived risks associated with revealing their HIV-status, and reported harassment of individuals living with HIV in Brazil [13].

In our study, some participants chose not to disclose their HIV diagnosis after facing negative repercussions from sharing their homosexuality or serological status with individuals they had considered close. These experiences included explicit or insidious rejection and blame or were influenced by the anticipation of adverse reactions from friends and family. Such reactions were often tied to religious beliefs that equate homosexuality with “immorality” and “promiscuity” and view HIV as a punishment for “sexual deviance”.

In Brazil, the impact of Catholic and Evangelical narratives on homosexuality and HIV is unmistakable [36,37]. These two religions, which together represent the beliefs of over 80% of the population, are the most widespread in the country, and ultraconservative Evangelical factions have been proliferating rapidly in recent years [36,37]. Despite individual variations among the different branches of Catholic and Evangelical churches, a common thread is a belief that homosexuality equates to immorality and sin and that HIV serves as punishment for those who defy the presumed heteronormative natural order [36,37].

In the early 1990s in Brazil, the initial diagnoses of HIV infection among young gay men reinforced stigmatizing beliefs, lending credence to the misconception that HIV exclusively affects this demographic. Terms such as “gay cancer” and “gay plague” became widespread in the country [38]. These perceptions appear to persist in Brazil. For instance, a qualitative study examined the psychosocial impact of disclosing an HIV diagnosis on 36 heterosexual men from a southern Brazilian capital. The researchers reported that surprise was a common reaction among participants, who had previously believed HIV to be a condition solely associated with gay men [19].

In our study, we confirmed the relationship between homophobia and serophobia within the religious context through subtle indications in the narratives of certain participants. For instance, participant P7, an active member of the Catholic Church engaged in volunteer services, noted that there were “reports” within the church regarding his sexual orientation. This likely reflects the participant’s perception of sexual orientation as a sin, crime, deviation, or misdemeanor. P7 also indicated that while church members might tolerate his sexual orientation, they would not extend the same tolerance to a gay man with HIV.

Regarding the physical health impact on the interviewees, seven reached the most advanced stage of HIV infection and were diagnosed with AIDS and various physical complications. This finding indicates that despite efforts and public investments in recent decades to detect HIV infection at earlier stages, which are associated with better prognoses, the challenge persists in Brazil. Indeed, many participants admitted to not undergoing HIV screening tests as frequently as recommended, and three disclosed that they had never taken an HIV test before their diagnosis.

Six of the seven participants diagnosed in the AIDS phase overcame the physical complications related to opportunistic diseases through hospitalization, intensive care, adherence to antiretroviral treatment, and self-care measures. However, for participant P1, who was diagnosed with AIDS and a critically low TCD4 lymphocyte count in 2020, the late-stage diagnosis and the inability to recover the immune system, even after achieving an undetectable viral load, signified a life-altering shift. P1 expressed this change, as follows:

*“Now I only have the present. I am not sure of anything. It is like living on an eternal tightrope. I see myself as a time bomb. I don’t know when I’m going to explode”.*

P1 also reported financial losses, loneliness, pain from invasive medical procedures, physical and emotional fatigue, lack of energy, and a fear of dying, all of which were described as profound distress.

The relationship between HIV-related stigma and delayed diagnosis was examined through a systematic review and meta-analysis [39]. This analysis synthesized findings from five studies in low- and middle-income countries, including Brazil. The data suggested that individuals with a heightened perception of serophobia had a twofold increased likelihood of receiving a late HIV diagnosis, specifically during the AIDS stage [40]. We, therefore, consider it plausible that the high perception of serophobia in the community and internalized serophobia, as verified among those interviewed in this study, are related to the late diagnosis of seven of the fifteen participants. Studies indicate that both serophobia and homophobia, whether internalized or perceived in the community, increase the likelihood of gay men engaging in high-risk sexual behaviors for HIV exposure [40,41]. They also decrease the likelihood of individuals undergoing regular testing [42,43] and adhering to treatment among those already diagnosed [44,45]. These three effects of stigma, in their various forms, partially explain the disproportionate increase in the incidence and prevalence of HIV among gay men in Brazil [3,4,5,6], including among those diagnosed late. Thus, we believe that developing and evaluating evidence-based interventions aimed at both the general population and health professionals is crucial for reducing stigma related to homosexuality and HIV. Such interventions should aim to enhance the quality of life for this population, decrease mental health issues associated with minority stress, and facilitate the early identification and treatment of cases. This approach remains underutilized in Brazil, yet it could significantly improve the response to the AIDS epidemic by achieving the UNAIDS 2023 targets: identifying 95% of HIV cases, ensuring that 95% of those diagnosed adhere optimally to treatment, and making the viral load undetectable in 95% of cases [46].

All participants reported a decline in mental health and quality of life in the months following their diagnosis, with 10 considering life more difficult and harboring less hope, even many months post-diagnosis. Three individuals disclosed suicidal ideation in the years after receiving their HIV diagnosis. These findings suggest that the adverse effects of living with HIV in Brazil may be chronic, extending beyond the initial months after diagnosis. This indicates an increased risk of developing mental disorders or exacerbating preexisting conditions. The deterioration of mental health indicators post-HIV diagnosis among gay men has been documented in multiple studies [31,47]. Consequently, social stigma and systematic discrimination related to sexual orientation and HIV status contribute to a stressful social environment marked by harmful events adversely affecting the mental health of our interviewees. The minority stress model posits that psychological stress stemming from HIV-related stigma and discrimination is linked to mental health disorders [28].

In our study, participants commonly reported social isolation, loneliness, and lack of social support. Therefore, we also suggest evidence-based interventions aimed at gay men living with HIV with a focus on developing social connections with others in the same condition. This measure has already been adopted by individual initiatives on social networks, in which people living with HIV talk about their diagnosis and living with the virus, and welcome newly diagnosed individuals. However, there is a lack of evidence of the possible beneficial effects of these initiatives, which could be evaluated in future quantitative and qualitative studies. We believe that the contact of recently diagnosed with HIV individuals with similar people who have already developed effective coping mechanisms to face possible difficulties related to living with HIV, whether physical or psychosocial in nature, is a promising measure, with probable potential to mitigate the effects of homophobia and serophobia on their mental health.

### Strengths, Limitations, and Future Directions

We believe we have achieved our study’s objectives by thoroughly understanding the experiences of Brazilian gay men living with HIV, encompassing both the diagnostic experience and its lifelong repercussions. Brazilian gay men are, epidemiologically, a highly relevant population for confronting the HIV/AIDS epidemic, given that they are disproportionally affected by HIV. However, the psychosocial impacts related to the diagnosis and aspects of living with HIV in Brazil have been little explored in depth in qualitative studies. Most qualitative research investigating the biopsychosocial impacts of HIV diagnosis and its repercussions has been carried out in developed countries, leaving especially Latin American and African gay men with HIV and their needs underrepresented. Thus, we believe that our study contributes to filling this representation gap.

Furthermore, we consider that the clinical interviews reached a significant level of depth and were able to produce original elements, which contribute to the understanding of the peculiarities of living with HIV among Brazilian gay men. Phenomena such as perceived and internalized homophobia, in addition to serological stigma, including when interacting with the multidisciplinary health professional teams of specialized HIV/AIDS services have been corroborated with previous research, including increased social isolation, reduced quality of life, and worsening of mental health indicators after HIV diagnosis. Points of interest regarding new themes, such as improvement in physical health, gratitude for the scientific advances in the area of HIV/AIDS, and the quality of care provided by the Brazilian health system, were mentioned by many participants, with these last two categories of analysis, as far as we know, being new contributions, thus requiring future research.

Hence, we believe that our study is relevant and unique. However, it is not without limitations. Online contact with participants precluded the confirmation of HIV diagnoses and the validation of clinical information, such as viral load and immunological status. Our participants were selected from a previous quantitative study in which seed participants nominated gay men known to be living with HIV. After completing the quantitative protocol, these individuals consented to participate in an interview, during which they disclosed their sociodemographic and clinical data to the interviewer. This introduces a significant selection bias, as we likely did not reach individuals whose sexual orientation and HIV-positive status are not publicly known. We suspect that among these individuals, the level of suffering from the compounded stigma and absence of a social support network may lead to even more significant psychosocial repercussions than those reported in our study.

Furthermore, because the study was conducted online, it digitally excluded gay men living with HIV who were unable to participate. It is important to conduct new qualitative studies that give voice to these individuals, as such research will uncover the dynamics of stigma concerning their unique particularities and individual experiences within this specific group. Additionally, relying solely on self-report data from interviews might not capture the full range or depth of experiences related to HIV diagnosis and its impacts because participants may not be consciously aware of more subtle or less predictable manifestations of the vicissitudes of living with HIV, or they may resist reporting them in the context of a single clinical interview.

Moreover, the cross-sectional nature of our qualitative study produced evidence of the experiences of Brazilian gay men living with HIV at a specific point in time, which may not have fully captured the dynamic nature of coping with an HIV diagnosis over time. Longitudinal data collection could provide deeper insights into the evolving biopsychosocial impacts and coping mechanisms of individuals living with HIV.

The sociodemographic and clinical profiles of participants in this research were diverse. We interviewed individuals with high, medium, and low education and income levels. Our participants ranged from those recently diagnosed with HIV to individuals who have lived with the virus for over 20 years. They included people diagnosed during the acute phase of HIV infection, who exhibited no significant clinical manifestations, as well as those diagnosed late, during the AIDS phase, with compromised immune systems and life-threatening diseases. Our axes were broad yet consistently analyzed in depth, encompassing various aspects of the biopsychosocial effects of living with HIV among Brazilian gay men. 

We recognize the importance of new qualitative studies focusing on Brazilian gay men with HIV that delve into the nuances of experiences according to each profile examined. This is particularly crucial for men who, despite achieving an undetectable viral load, have not experienced adequate immunological recovery and remain in the AIDS phase. Giving these individuals a voice to express their experiences and the suffering associated with physical illness is of utmost importance. Additionally, investigating the mechanisms behind late diagnosis is relevant, as it enables understanding from the perspective of those affected, potentially offering fresh insights for healthcare teams and policymakers in public health to prevent new cases of late diagnosis.

Brazil is a country of continental dimensions, characterized by significant regional differences in culture, customs, income, education, and other psychosocial indicators. In our study, most participants were graduated, young, and white. Therefore, generalizations of our results should be made with caution because our sample is relatively small and does not necessarily represent the sociodemographic profile of the Brazilian population, which is mainly composed of brown and black people with low education levels. We believe it is especially important, in future studies, to explore the intersections of multiple forms of stigma, such as racism, classism and their relationship with homophobia and serological stigma. This enables a deeper understanding of the effects of the overlapping of different forms of social marginalization and produces knowledge that can support public care policies for this key population in tackling the HIV/AIDS epidemic.

## 5. Conclusions

Our study provided a comprehensive understanding of Brazilian gay men’s experiences living with HIV, focusing on the moment of diagnosis and its biopsychosocial impacts. The findings underscore that the HIV diagnosis was often perceived as a potentially traumatic event, particularly when coupled with inadequate reception and emotional support from healthcare professionals. Participants reported a decline in mental health following their diagnosis, regardless of whether they experienced physical health consequences related to HIV. This decline was attributed to the compounded stigma of being both homosexual and HIV-positive, whether internalized or perceived within the community. Such stigma was linked to withdrawal from support networks and reluctance to pursue affective-sexual relationships. Common experiences among participants included guilt, fear associated with the discovery of their HIV status, feelings of social isolation and loneliness, perceived lack of social support, and detrimental effects on affective-sexual relationships.

More than 40 years after the HIV epidemic began, widespread perceptions of homophobia and serophobia among gay men living with the virus were reported by Brazilian participants. These attitudes were even present in the multidisciplinary teams of specialized services, indicating that stigma associated with homosexuality and HIV has not evolved in step with the significant biomedical advances in diagnosing and treating the infection, particularly in Brazil. Consequently, there is a dual challenge, which includes developing mental health care policies for gay men living with HIV who face a compounded stigma and creating and consistently implementing public policies that educate the Brazilian population about HIV and promote respect for sexual diversity.

## Figures and Tables

**Table 1:** Sociodemographic and clinical description of research participants.

ID	Age	Race	Education	Occupation	Income ^1^	Marital Status	Diagnosis Time ^2^	AIDS	Current Health Status
P1	36	White	Master’s degree	Public employee on leave	2	Single	2.5	YES	S *
P2	35	Brown	Bachelor’s degree	Researcher (Master’s Scholarship)	2	Single	7.2	YES	A **
P3	52	Black	High school	Merchant	2.5	Single	23.1	NO	A
P4	35	White	Doctorate degree	Biomedical and Postgraduate Professor	20	Single	2.5	NO	A
P5	32	Black	Vocational/technical education	Technical Analyst in the private sector	2	Stable union	4.3	NO	A
P6	43	Brown	Bachelor’s degree	High school teacher	5.5	Stable union	3.0	NO	A
P7	39	White	Master’s degree	Pharmacist and undergraduate professor	7.5	Single	2.5	NO	A
P8	31	Brown	High school	Secretary in the private sector	1	Single	2.1	YES	A
P9	43	White	Elementary School	Self-employed hairdresser	2.5	Single	8.9	NO	A
P10	39	White	Bachelor’s degree	Freelance advertiser	7	Single	2.1	YES	A
P11	46	White	Master’s degree	Private sector lawyer	8	Single	8.4	YES	A
P12	24	White	Master’s degree	Private sector lawyer	5	Dating	0.0	NO	A
P13	34	White	Master’s degree	Engineer and entrepreneur	10	Stable union	2.0	NO	A
P14	62	White	Bachelor’s degree	Merchant	8	Stable union	26.6	YES	S
P15	29	White	High school	Waiter and Merchant	2	Single	12.6	YES	A

^1^ The minimum wage in the first half of 2022, the year in which participants were interviewed, was R$1212.00 = +/−220€/245$USD; ^2^ Time presented in years; * S = Symptomatic; ** A = Asymptomatic.

## Data Availability

Data will be available upon request.
